# Effects of unilateral training on rapid force production in athletes: a systematic review and meta-analysis

**DOI:** 10.3389/fphys.2026.1805250

**Published:** 2026-04-21

**Authors:** Yuan Zhuang, Yugang Zhang, Lei Ma, JoonYoung Han

**Affiliations:** 1 School of Physical Education, Yeungnam University, Daegu, Republic of Korea; 2 Department of Physical Education, Yuncheng University, Yuncheng, China

**Keywords:** 10-m sprint performance, 5-msprint performance, athletes, countermovement jump (CMJ) performance, rapid ballistic force production, unilateral training

## Abstract

**Introduction:**

Unilateral training has been increasingly applied in athletic conditioning; however, its effects on rate of force development (RFD) remain unclear. This study aimed to systematically evaluate the effects of unilateral training on RFD in athletes.

**Methods:**

A systematic search was conducted across major electronic databases to identify studies examining the effects of unilateral training on RFD. Eligible studies were included based on predefined criteria, and a meta-analysis was performed to quantify the overall effects.

**Results:**

The findings indicated that unilateral training produced significant improvements in RFD among athletes, although the magnitude of effects varied depending on training protocols and participant characteristics

**Discussion:**

Unilateral training appears to be an effective strategy for enhancing RFD in athletes. These findings provide practical implications for strength and conditioning programs, while highlighting the need for further research to clarify optimal training parameters.

**Systematic Review Registration:**

https://www.crd.york.ac.uk/prospero, identifier CRD420251030088.

## Introduction

Athletic performance is generally considered to be the result of multiple interacting factors, including innate athletic talent as well as the technical, tactical, and physical capacities developed through systematic training (Smith, 2003). Evaluating these factors not only enables coaches to better understand the specific requirements of a given sport, but also helps clarify training priorities for athletes, thereby allowing the design of targeted training programs aimed at improving sport-specific skills (Den Hartigh et al., 2018). Among these factors, physical fitness represents a fundamental prerequisite for athletic performance (Cureton, 1956). Physical fitness constitutes the basic capacity required for competitive performance and is formed through the coordinated integration of strength, speed, flexibility, and muscular power. Together, these elements support the execution of sport-specific techniques and the physiological demands of competition, thereby contributing to the attainment of optimal performance outcomes (Liu and He, 2022). Among the various components of physical fitness, explosive performance is a key ability that allows athletes to produce force rapidly and execute high-intensity movements efficiently within a short time frame. Its level partly determines the quality of performance in high-level competitions and influences the likelihood of achieving competitive success (Cronin and Sleivert, 2005). In volleyball, for example, explosive jumping ability is a critical factor influencing the efficiency of net play. Through rapid muscle fiber recruitment and optimization of the stretch–shortening cycle, it directly contributes to greater vertical height during spiking and blocking, thereby facilitating tactical execution (Keoliya et al., 2024). Similarly, in basketball, training interventions targeting explosive performance have been shown to improve athletes’ performance outcomes (Huang et al., 2023). Therefore, adopting appropriate training strategies to enhance sport-specific explosive performance is essential for optimizing athletes’ performance in competition.

In daily training practice, the importance of improving explosive performance for athletic performance and competitive advantage has been widely recognized (Aksović et al., 2021; Wang et al., 2023). Consequently, extensive research has explored different training approaches aimed at developing explosive performance, including resistance training (Santos and Janeira, 2012), complex training (Santos and Janeira, 2008), high-intensity interval training (Fajrin and Kusnanik, 2018), and unilateral training (Ramírez-Campillo et al., 2015). Among these methods, unilateral training refers to strength exercises performed predominantly with a single limb. Typical forms include unilateral lower-limb strength exercises and loaded squats performed under unilateral conditions. By enhancing force production and control on each side of the body, unilateral training may improve limb function and overall athletic performance (Kilinç et al., 2009). Such training is frequently applied in specific contexts, including postural development in youth athletes (Kilinç et al., 2009 and sports rehabilitation programs (Rogalski et al., 2025). Unilateral training is characterized by asymmetric loading and single-limb movement patterns. During unilateral force production, the body must actively regulate overall stability and inter-limb coordination. The underlying mechanism involves not only strengthening the force output of individual muscle groups but also activating core musculature responsible for anti-flexion and anti-rotation control, thereby maintaining movement integrity during task execution. Furthermore, asymmetric loading may stimulate neuromuscular adaptations across the kinetic chain and enhance the efficiency of force transmission. Through these mechanisms, unilateral training may facilitate improvements in rapid force production and consequently enhance explosive performance (Munn et al., 2005). In practical training settings, coaches often integrate unilateral training with sport-specific training strategies and athletes’ individual characteristics in order to maximize improvements in explosive performance (Moran et al., 2021). In other words, the application of unilateral training should follow the principle of aligning training methods with both the characteristics of the sport and the individual athlete. For instance, training programs combining unilateral training with plyometric exercises have been used to improve explosive performance and physical performance in soccer players (Ramírez-Campillo et al., 2015). Similarly, unilateral strength training programs have been implemented to enhance limb symmetry in soccer athletes (Bettariga et al., 2023a). These findings suggest that the effectiveness of unilateral training may vary depending on the specific sport and the characteristics of the athletes involved.

Previous research has shown that under certain conditions a phenomenon known as the bilateral deficit may occur when both limbs produce force simultaneously, meaning that the sum of unilateral force outputs may exceed the force produced during bilateral contractions (Makaruk et al., 2011; Fountaine, 2018). Compared with traditional bilateral training, the potential advantages of unilateral training include several aspects: (a) inducing delayed neural adaptations in the contralateral limb (Mastalerz and Sadowski, 2020); (b) improving load perception and metabolic responses (Costa et al., 2015); and (c) enhancing peak muscle force and rate-related performance variables (Adamson et al., 2008). These adaptations are closely associated with improvements in explosive performance indicators such as muscle strength (Speirs et al., 2016) and movement velocity (Muirhead et al., 2024). Indeed, athletes with superior explosive performance often demonstrate higher levels of competitive performance (Aoki et al., 2015). For example, to efficiently perform complex gymnastics movements such as the vault, athletes must develop substantial explosive performance in both the upper and lower limbs (Farley et al., 2020). Similarly, Zhao et al. (2023) reported that the development of explosive performance plays a crucial role in enhancing performance in rugby players. These findings collectively highlight the potential importance of unilateral training for improving athletes’ explosive performance.

Conceptually, unilateral training refers to a training approach in which a single limb serves as the primary load-bearing or control condition during exercise. Its key characteristic lies in modifying traditional bilateral movement patterns and emphasizing maximal force production and neuromuscular coordination under asymmetric conditions (Vandervoort et al., 1984). This training mode typically requires athletes to complete movements under single-limb dominance, which imposes greater demands on coordination and postural stability, thereby providing a highly specific training stimulus (Espada et al., 2023). From a movement perspective, unilateral training is often integrated with other training modalities and involves multi-joint and multi-planar movement patterns. Such compound training structures incorporate several key elements, including strength, flexibility, and stability control (Liu et al., 2025). Consequently, unilateral training may not only provide targeted stimulation to specific limbs but also contribute to improvements in overall athletic performance. Although numerous experimental studies have investigated the effects of unilateral training on athletic performance, and several reviews have summarized this body of literature, the scientific evidence regarding its benefits for explosive performance remains limited. Therefore, the present systematic review aims to clarify the effects of unilateral training on explosive performance in athletes, with the goal of providing scientific evidence to inform training strategies designed to improve explosive performance.

## Methodology

### Protocol and registration

This systematic review and meta-analysis followed the PRISMA Statement for data screening, extraction, and analysis (Moher et al., 2009). The review protocol was prospectively registered in the PROSPERO. The registration can be accessed at https://www.crd.york.ac.uk/prospero (Registration No. CRD420251030088).

### Eligibility criteria

The PICOS framework (Population, Intervention, Comparator, Outcomes, Study design) was used to evaluate the eligibility of the included studies (McKenzie et al., 2019). The specific criteria were as follows.

Population: Participants were healthy male and female athletes. All age groups were eligible, and no restrictions were placed on competitive level or sport discipline.
Intervention: The unilateral training intervention lasted for at least four weeks. The training program had to clearly involve exercises performed primarily with one limb (left or right) and be designed to develop sport-related movement abilities. Training could target the upper limbs, lower limbs, or both. The training content included, but was not limited to, unilateral resistance exercises such as single-leg squats, split squats, and single-arm rows, as well as balance and stability exercises. The core feature of these exercises is the use of unilateral limb loading to improve inter-limb strength balance and enhance neuromuscular control of the core (Pori et al., 2023). To clearly evaluate the effects of unilateral training, studies that combined unilateral training with conventional bilateral training without a distinct comparison group were excluded. However, studies that included a clearly defined unilateral training group and a comparison group performing bilateral training with equivalent training volume were eligible for inclusion.
Comparison: The control group performed conventional training with the same intensity and volume as the intervention group but did not include any unilateral training exercises.
Outcomes: The study had to report at least one outcome related to the effect of unilateral training on explosive performance. These outcomes were defined as the ability to generate high force or velocity within a short period and were typically assessed using sport-specific explosive tasks such as jump or sprint performance. Eligible studies were required to report the effects of unilateral training on these outcomes or provide pre- and post-intervention values. Studies that assessed only general strength measures (e.g., maximal isometric strength) or endurance without reflecting explosive performance (e.g., sprint speed) were excluded.
Study design: Only randomized controlled trials were included in this review.

Studies that did not meet the above inclusion criteria were excluded.

### Search strategy and selection process

On August 30, 2025, four electronic databases were systematically searched to identify relevant studies: China National Knowledge Infrastructure (CNKI), Web of Science, PubMed, and EBSCOhost. During the search process, a previous review (Moran et al., 2021) was consulted to help develop the search strategy. When searching these databases, search terms and Boolean operators were applied using both individual and combined search terms (see **Table 1**). The search strategy included the following terms and operators: (“Athletes”[Mesh]) OR ((athlete*[Title/Abstract]) OR (player*[Title/Abstract])) AND ((unilateral training[Title/Abstract]) OR (single-leg training[Title/Abstract]) OR (unilateral exercise[Title/Abstract]) OR (single-limb training[Title/Abstract]) OR (asymmetrical training[Title/Abstract])) AND ((power[Title/Abstract]) OR (jump[Title/Abstract]) OR (sprint[Title/Abstract]) OR (performance[Title/Abstract])). In addition, to identify potentially relevant studies that might not have been captured in the initial search, previously published review articles (Moran et al., 2021; Manca et al., 2017) published before August 30, 2025 were examined. Google Scholar was also searched to identify **Supplementary Materials** and additional relevant studies. Furthermore, the reference lists of the included articles were manually screened to identify studies that may not have been retrieved in the initial database search. Finally, to ensure the completeness and accuracy of the literature search and study identification process, a professional librarian with relevant expertise was invited to review and verify the entire search procedure.

**Table 1:** PEDro scale scores of the included studies.

Study name	N1	N2	N3	N4	N5	N6	N7	N8	N10	N11	Total	Study quality
Deng et al. (2025)	1	1	0	1	0	0	0	0	1	1	5	Moderate
Núñez et al. (2018)	1	1	0	1	0	0	0	1	1	1	6	High
Stern et al. (2020)	1	1	0	1	0	0	0	1	1	1	6	High
Ramírez-Campillo et al. (2015)	1	1	0	1	0	0	0	1	1	1	6	High
Bettariga et al. (2023b)	1	1	1	1	0	0	0	1	1	1	6	High
Zhao et al. (2024)	1	1	1	1	0	0	1	1	1	1	7	High
Cao et al. (2024)	1	1	1	1	0	0	1	1	1	1	7	High
Bettariga et al. (2023b)	1	1	1	1	0	0	0	0	1	1	5	Moderate
Drouzas et al. (2020)	1	1	0	0	0	0	0	0	1	1	3	Low
Belegišanin et al. (2025)	1	1	0	0	0	0	0	1	1	1	4	Moderate
Gonzalo-Skok et al. (2022)	1	1	0	0	0	0	0	1	1	1	3	Low
Zhang et al. (2024)	1	1	0	0	0	0	0	1	1	1	4	Moderate
Ramirez-Campillo et al. (2018)	1	1	0	0	0	0	1	1	1	1	5	Moderate
Gonzalo-Skok et al. (2019)	1	1	0	1	0	0	1	0	1	1	5	Moderate
Shi and Wu (2019)	1	1	0	1	0	0	0	1	1	1	5	Moderate
Fisher and Wallin (2014)	1	1	1	1	0	0	0	0	1	1	5	Moderate
Gonzalo-Skok et al. (2017)	1	1	0	1	0	0	0	0	1	1	4	Moderate
Speirs et al. (2016)	1	1	0	1	0	1	1	0	1	1	7	High

All included studies were assessed using the PEDro scale, which comprises 11 methodological criteria. Each item is scored as either “satisfied” (1 point) or “not satisfied” (0 points), yielding a maximum total score of 10 points (Item 1 is not included in the total score). The PEDro scale is used to quantify the methodological rigor and internal validity of each study.

**Figure 1:**
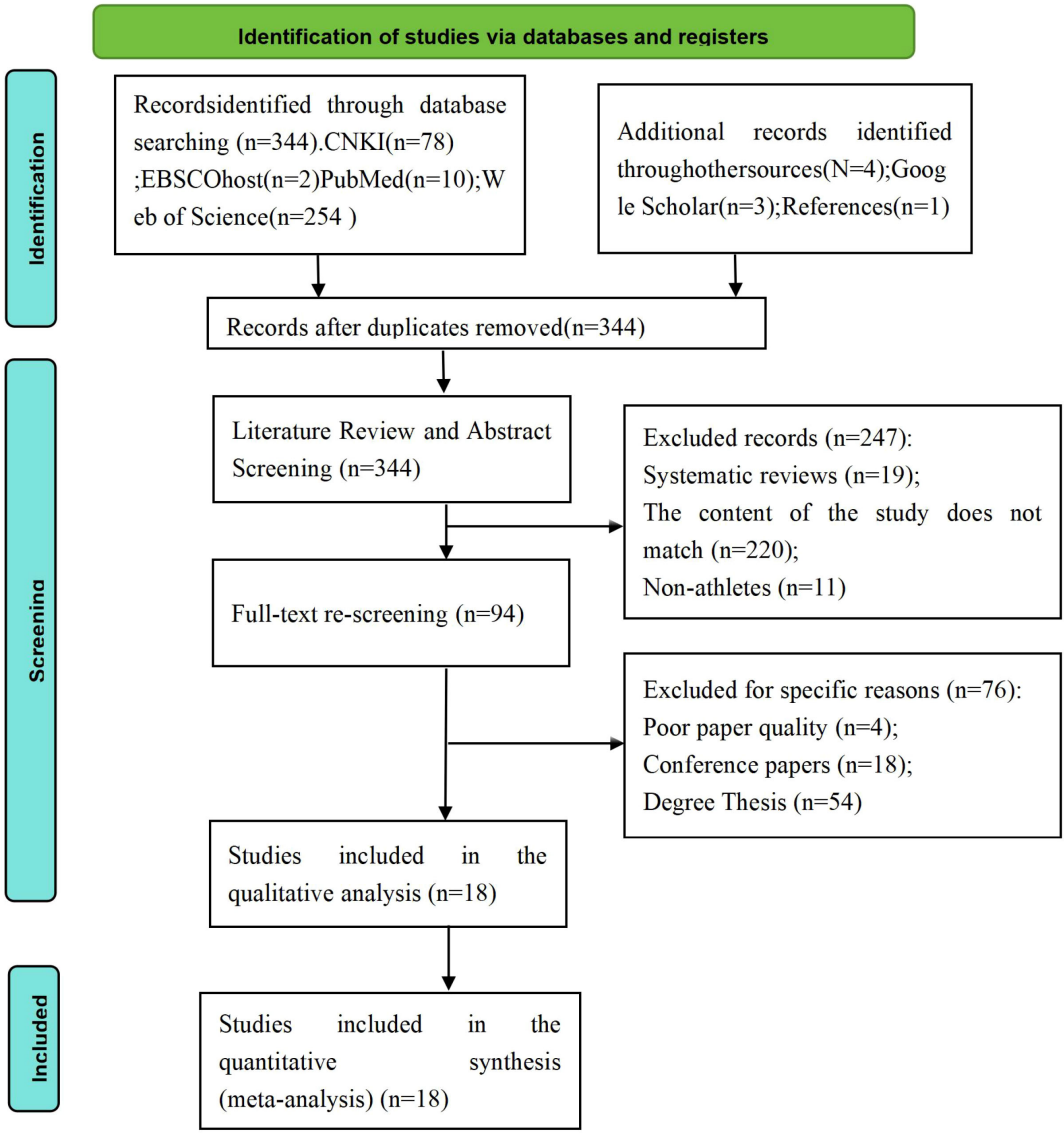
PRISMA flow diagram.

The literature search and screening process consisted of four main steps (**Figure 1**). First, duplicate records were removed. Second, studies were excluded by screening titles and abstracts if they were systematic reviews, involved non-athlete populations, or did not match the target research focus. Third, full texts were assessed to exclude low-quality studies, as well as theses and conference proceedings. Fourth, the remaining articles were assessed for eligibility to determine the final set of studies included in the analysis. The entire screening process was independently conducted by two reviewers (ZY and ZYG). In cases of disagreement during the screening process, a third reviewer (ML) was consulted for further discussion until consensus was reached.

### Data extraction

Two reviewers (ZY and ML) independently extracted study information using Microsoft Excel spreadsheets, and the extracted data were subsequently verified by a third reviewer (ZYG). The following information was extracted from each included study:

- general study information, including the first author, year of publication, and country;
- participant characteristics, including age, sex, sport, sample size, competitive level, and training experience;
- characteristics of the unilateral training intervention, including training duration, frequency, and session length;
- outcome measures related to explosive performance;
- the mean values and standard deviations of each outcome measure for both the intervention and control groups before and after the intervention;
- methodological information of the included studies, including the reporting of randomization procedures, allocation concealment, and blinding of outcome assessors, which were used for methodological quality assessment and for grading the certainty of evidence using the GRADE approach.

### Study quality assessment

To assess the methodological quality of the included studies, two reviewers (ZY and ML) independently evaluated each study using the PEDro scale. The PEDro scale has demonstrated good validity (De Morton, 2009) and reliability (Maher et al., 2003) and is considered a reliable tool for assessing the methodological quality of included studies. The assessment results were cross-checked by a third reviewer (ZYG), and final agreement was reached among all three reviewers. The PEDro scale uses a scoring system ranging from 0 to 10. A score of ≤3 indicates low methodological quality, 4–5 indicates moderate quality, and 6–10 indicates high methodological quality. The scale consists of 11 methodological assessment items. Each item that meets the criteria receives one point, except for Item 1 (external validity), which is not included in the total score. In addition to the overall quality score, this study also recorded PEDro items related to the risk of bias, including the reporting of randomization procedures, allocation concealment, and blinding of outcome assessors. These indicators were used to further evaluate the potential risk of bias of the included studies and its possible influence on the study results. During the assessment process, if disagreements occurred between the two reviewers, the third reviewer (ZYG) participated in further discussion until consensus was reached.

### Certainty of evidence

To minimize subjective bias and improve the objectivity of the evaluation results, two reviewers (ZY and ML) independently assessed the certainty of evidence for each outcome using the internationally recognized Grading of Recommendations Assessment, Development and Evaluation (GRADE) framework. The certainty of evidence was classified into four levels: high, moderate, low, and very low (Guyatt et al., 2011).

All included studies were initially considered to provide high-quality evidence, and the certainty level was subsequently downgraded according to the following criteria:

- Risk of bias. If the median PEDro score of the included studies was lower than 6, the certainty level was downgraded by one level.
- Indirectness. As the included studies clearly defined the population, intervention, comparator, and outcome measures, the risk of indirectness was considered low; therefore, no downgrading was applied for this criterion.
- Publication bias. Potential publication bias was evaluated based on the literature search strategy. If a risk of publication bias was identified among the included studies, the certainty level was downgraded by one level.
- Inconsistency. If substantial statistical heterogeneity was observed (I²>75%), the certainty level was downgraded by one level.
- Imprecision. This was evaluated based on sample size adequacy and the precision of the effect estimates. If the total sample size was fewer than 800 participants or the direction of the 95% confidence interval of the effect estimate was unclear, the certainty level was downgraded by one level. If both conditions were present, the certainty level was downgraded by two levels (Ramirez-Campillo et al., 2022).

If the number of included studies was insufficient to perform a meta-analysis, the certainty of evidence for that outcome was directly rated as very low. Outcomes that could not be quantitatively synthesized through meta-analysis were considered to have fundamental limitations and were therefore also rated as very low certainty. In addition, inconsistencies between methodological procedures and reporting in areas such as randomization, allocation concealment, and blinding may increase the risk of bias and consequently reduce the certainty of evidence.

Based on the overall GRADE assessment, the certainty of evidence for the included studies in this review ranged from low to moderate, suggesting that the findings regarding the effects of unilateral training on explosive performance in athletes should be interpreted with caution.

### Statistical analysis

In the field of physical therapy (PT), research samples are typically small (Ramirez-Campillo et al., 2022). Considering this situation, although methodologically it is permissible to conduct comparative analyses with only two studies (Valentine et al., 2010), a more conservative inclusion criterion was adopted in the present study. Specifically, a meta-analysis was performed only when three or more studies were available (Deng et al., 2023). To calculate the effect size, the mean values and standard deviations of indicators related to explosive performance measured before and after the intervention were extracted, and the results were standardized based on the pre- and post-intervention data. When the required data could not be obtained directly from the published text, for example when results were presented only in graphical form, attempts were made to contact the corresponding authors to obtain the original data. To ensure complete data acquisition, for studies in which results were reported only in figures, pre- and post-intervention values were extracted from images using WebPlotDigitizer software (version 4.5). The reliability of this tool has been previously confirmed (r=0.99, p<0.001) (Drevon et al., 2017). In the meta-analysis, a random-effects model was applied, and study weights were calculated using the inverse-variance method to pool the effect sizes. This model is appropriate for synthesizing studies with potential heterogeneity, as it assigns weights based on the inverse of the standard error of each study and incorporates the true between-study variability into the effect estimation (Kontopantelis et al., 2013). Consequently, this model typically produces wider confidence intervals, thereby providing more conservative and generalizable estimates. All effect sizes were reported with 95% confidence intervals. To evaluate the practical significance of the results, the following standardized mean difference thresholds were adopted: <0.2 (trivial), 0.2–0.6 (small effect), 0.6–1.2 (moderate effect), 1.2–2.0 (large effect), 2.0–4.0 (very large effect), and >4.0 (extremely large effect) (Hopkins et al., 2009). In addition, for studies including multiple intervention groups, the control group sample was proportionally divided to allow comparisons across all participants (Deng et al., 2023). Heterogeneity among the included studies was assessed using the I² statistic, with values <25% indicating low heterogeneity, 25–75% indicating moderate heterogeneity, and >75% indicating high heterogeneity (Higgins and Thompson, 2002). Publication bias was evaluated using the extended Egger test, and if significant bias was detected, a sensitivity analysis was conducted. All statistical analyses were performed using Comprehensive Meta-Analysis software (version 3.0), and the level of statistical significance was set at p<0.05.

## Results

### Study selection

As shown in **Figure 1**, a total of 344 records were initially identified through database searches, with an additional 4 studies identified through Google Scholar and reference list screening. After manual removal of duplicates, 340 records remained. Following title and abstract screening, 92 articles were retained for full-text assessment. Ultimately, 18 studies met the inclusion criteria and were included in the meta-analysis.

### Study quality assessment

Two researchers (ZY and ML) independently assessed the methodological quality of the included studies using the PEDro scale (see **Table 1**). In cases of disagreement, a third author (ZYG) was consulted to resolve discrepancies until consensus was reached. In addition to the total PEDro score, specific methodological items reported in the included studies were recorded, including randomization procedures, allocation concealment, and assessor blinding. The results indicated that nine included studies obtained PEDro scores of 4–5, suggesting a moderate level of methodological quality. In addition, seven studies scored above 6, indicating relatively high methodological quality. However, two studies received a score of 3; therefore, these studies were excluded from the present review. It is noteworthy that some studies reported insufficient information regarding key methodological items such as randomization, allocation concealment, and blinding. This suggests that potential methodological bias may exist in the included studies, which could influence the interpretation of the meta-analysis results.

### Risk of bias assessment

**Figure 2:**
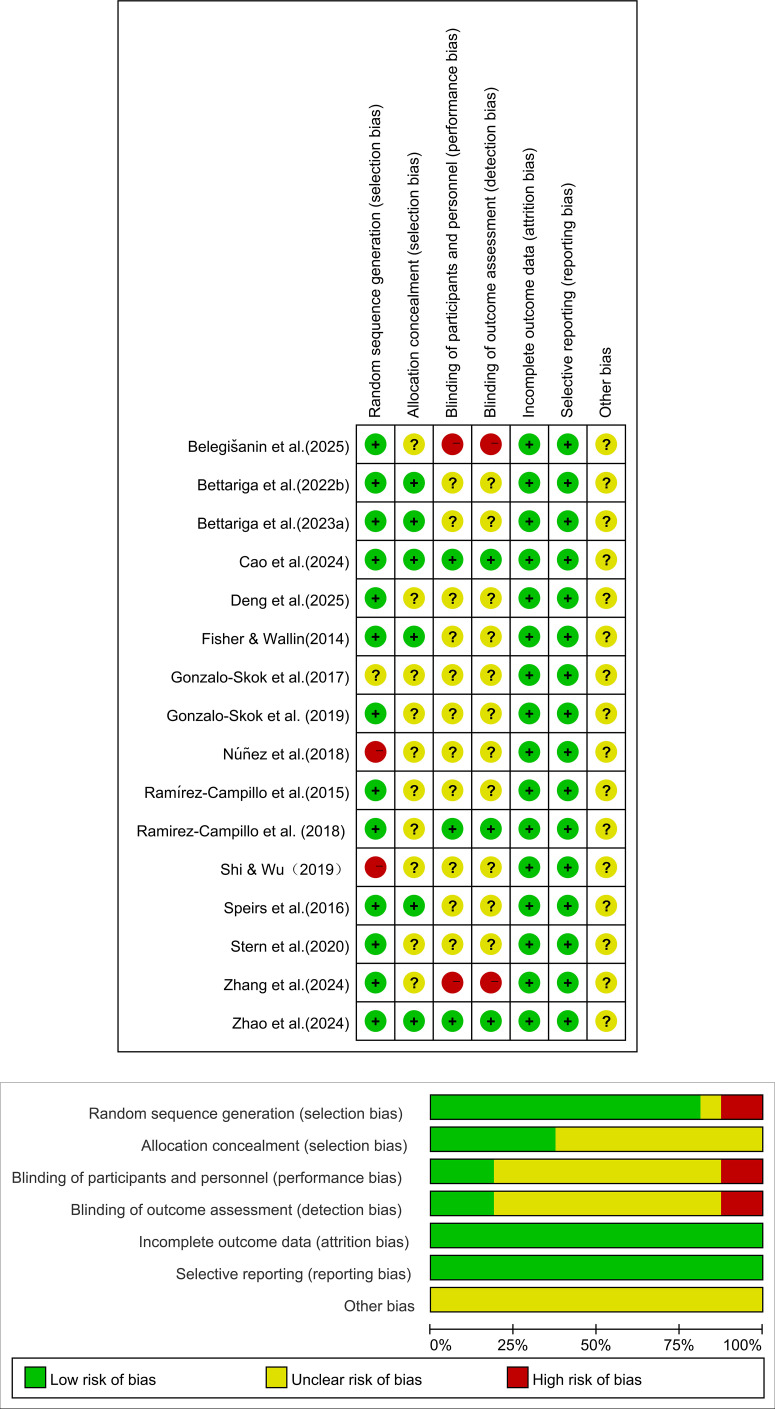
Risk of bias assessment.

Two researchers (ZYG and ML) independently assessed the risk of bias of the included studies in accordance with the guidelines of the Cochrane Collaboration, using RevMan Manager (version 5.4.1). Any disagreements were resolved through discussion with a third author (ZYG) until consensus was reached. Each included study was evaluated across seven domains of bias risk and classified as having a “low risk of bias,” “unclear risk of bias,” or “high risk of bias.” An overall judgment of the risk of bias for each study was then made (see **Figure 2**). Overall, most of the included studies were rated as having a low risk of bias in terms of random sequence generation. However, reporting on methodological procedures such as allocation concealment and blinding remained insufficient in several studies, leading some of them to be classified as having an unclear risk of bias. This indicates that certain limitations may still exist in the methodological design and reporting of the included studies, and these potential sources of bias may influence the interpretation of the meta-analysis results.

### Study characteristics

**Table 2** summarizes the intervention characteristics of participants in the randomized controlled trials (RCTs) included in the present study. A total of 17 studies met the inclusion criteria, including 16 English-language studies (Deng et al., 2025; Núñez et al., 2018; Stern et al., 2020; Ramírez-Campillo et al., 2015; Bettariga et al., 2023a; Zhao et al., 2024; Cao et al., 2024; Bettariga et al., 2023b; Belegišanin et al., 2025; Zhang et al., 2024; Ramirez-Campillo et al., 2018; Gonzalo-Skok et al., 2019; Shi and Wu, 2019; Fisher and Wallin, 2014; Gonzalo-Skok et al., 2017; Speirs et al., 2016) and one Chinese-language study (Shi and Wu, 2019). The publication years ranged from 2014 to 2025. In terms of study design, 13 studies adopted a two-group parallel design (Deng et al., 2025; Núñez et al., 2018; Stern et al., 2020; Bettariga et al., 2023b; Bettariga et al., 2023a; Belegišanin et al., 2025; Zhang et al., 2024; Ramirez-Campillo et al., 2018; Gonzalo-Skok et al., 2019; Shi and Wu, 2019; Fisher and Wallin, 2014; Gonzalo-Skok et al., 2017; Speirs et al., 2016), one study employed a three-group design (Zhao et al., 2024), and two studies used four-group designs (Cao et al., 2024; Ramírez-Campillo et al., 2015). Regarding geographical distribution, five studies were conducted in China (Deng et al., 2025; Zhao et al., 2024; Cao et al., 2024; Zhang et al., 2024; Shi and Wu, 2019), four in Spain (Núñez et al., 2018; Ramirez-Campillo et al., 2018; Gonzalo-Skok et al., 2019, 2017), four in the United Kingdom (Stern et al., 2020; Bettariga et al., 2023b; Fisher and Wallin, 2014; Speirs et al., 2016), one in Chile (Ramírez-Campillo et al., 2015), one in Italy (Bettariga et al., 2023a), and one in Serbia (Belegišanin et al., 2025).

**Table 2:** Characteristics of participants and PT interventions in the included studies.

Study	Country	Intervention	N	Sex	Age	Level/ experience	Sport	Comparison	Replace	Outcome(s)
Deng et al. (2025)	China	Freq: 2 times/ week Time: NR Length: 8 weeks	16	F	EG1:18.75 ± 0.707 EG2:19.25 ± 0.707	Collegiate > 5-yrs	Volleyball	EG1:Unilateral Training Group EG2:Bilateral Training Group	NO	CMJ↑ SLJ↑
Núñez et al. (2018)	Spain	Freq: 2 times/ week Time: NR Length: 6 weeks	27	M	EG1:22.8 ± 2.9 EG2:22.6 ± 2.7	Collegiate NR	Team sports	EG1:Unilateral Lunge Squat Group EG2:Bilateral Lunge Squat Group	NO	CMJ→
Stern et al. (2020)	UK	Freq: 2 times/ week Time: NR Length: 6 weeks	23	M	17.6 ± 1.2	Local soccer Team > 2-yrs	Soccer	EG1:Bulgarian split squat, single-leg vertical jump, single-leg drop jump, single-leg standing long jump. EG2:Back squat, bilateral vertical jump, bilateral drop jump, bilateral standing long jump.	NO	CMJ↓ RSI→ 10M↑
Ramírez-Campillo et al. (2015)	Chile	Freq: 2 times/ week Time: NR Length: 6 weeks	54	M	CG:1.2± 2.4 BG:11.0 ± 2.0 UG:11.6 ± 1.7 B + UG:11.6 ± 2.7	Sub-elite 3–4 yrs	Soccer	CG: Conventional Soccer Training EG1:Unilateral Concentric-Concentric Jump Training Group EG2:Bilateral Concentric-Concentric Jump Training Group EG3:Combined Bilateral+Unilateral Jump Training Group	YES	CMJ ↑ CMJ-R ↑ CMJ-L ↑
Bettariga et al. (2023a)	UK	Freq: 2 times/ week Time: 60min Length: 6 weeks	24	M	25.4 ± 4.9	Sub-elite >3 yrs	Soccer	CG: Conventional Training EG: Unilateral Strength+Plyometric Jump Training	NO	5 m→ 10 m↑
Zhao et al. (2024)	China	Freq: 3–4 times/ week Time: NR Length: 8 weeks	75	M	EG1:16.2 ± 0.6 EG2:16.4 ± 0.6 Control:16.4 ± 0.5	Local Basketball Players Team >3 yrs	Basketball	CG: Conventional Training EG1:Unilateral Plyometric Jump Training Group EG2:Bilateral Plyometric Jump Training Group	NO	CMJ↑
Cao et al. (2024)	China	Freq: 2 times/ week Time: NR Length: 8weeks	66	M	UT: EG1:15.9 ± 0.9 BT: EG2:6.3 ± 0.8 UBT: EG3::16.2 ± 0.7 Control:CG:16.1 ± 0.8	Local Basketball Players Team >2 yrs	Basketball	CG: Conventional Training EG1:Unilateral Plyometric Training Group EG2:Bilateral Plyometric Training Group EG3:Combined Unilateral+Bilateral Plyometric Training Group	NO	CMJ-L↑ CMJ-R↑
Bettariga et al. (2023a)	Italy	Freq: 2 times/ week Time: NR Length: 6weeks	24	M	EG:27.0 ± 4.8 CG:23.8 ± 4.8	Local soccer Team >3 yrs	Soccer	CG: Conventional Training EG: Unilateral Strength+Power Training	NO	CMJ-R ↑ CMJ-L↑ SLBJ-R ↑ SLBJ-L↑
Belegišanin et al. (2025)	Serbia	Freq: 4 times/ week Time: 90min Length: 6weeks	22	M	EG(UPT):15.5 ± 0.5 EG2(BPT):15.2 ± 0.4	Local soccer Team >4 yrs	Basketball	EG1:Unilateral Flywheel Strength Training Group EG2:Bilateral Flywheel Strength Training Group	NO	RSI↑ CMJ↑ 5m↑
Zhang et al. (2024)	China	Freq: 3 times/ week Time: 30min Length: 10weeks	30	M	EG:20.9 ± 1.1 CG:20.9 ± 0.9	Collegiate >4 yrs 4.1 ± 0.8 yrs	Basketball	CG: Conventional Training EG: Unilateral Combined Training	NO	CMJ ↑ SLJ↑ 10m→
Ramirez-Campillo et al. (2018)	Spain	Freq: 2 times/ week Time: NR Length: 8weeks	18	M	EG(UG):17.3 ± 1.1 CG(TG):17.6 ± 0.5	Collegiate >8 yrs	Soccer	CG: Conventional Training EG: Unilateral Plyometric Training Group	NO	CMJ↓
Gonzalo-Skok et al. (2019)	Spain	Freq: 2 times/ week Time: 35-40min Length: 6weeks	18	M	13.2 ± 0.7	National-level Athlete	Basketball	CG: Bilateral Vertical Power Training EG: Unilateral Horizontal Power Training	NO	CMJ↑ 5m↑ 10m↑
Shi and Wu (2019)	China	Freq: 3 times/ week Time: NR Length: 10weeks	16	F	20. 5	National-level Athlete >5yrs	Judo	CG: Bilateral Lower-Limb Resistance Training EG: Unilateral Limb Resistance Training	NO	CMJ ↑
Fisher and Wallin (2014)	UK	Freq: 2 times/ week Time: NR Length: 6weeks	15	M	CG(TG):20.14 ± 1.77 EG(UG):19.8 ± 1.49	collegiate level	Rugby	CG: Bilateral Lower-Limb Resistance Training EG: Unilateral Limb Resistance Training	NO	10m↓
Gonzalo-Skok et al. (2017)	Spain	Freq: 2 times/ week Time: NR Length: 6weeks	22	M	EG(UG):16.8 ± 1.7 CG(TG):16.7 ± 1.7	National-level Athlete >2 yrs	Basketball	CG: Bilateral Lower-Limb Resistance Training Group EG: Unilateral Limb Resistance Training Group	NO	CMJ→ 5 m→
Speirs et al., 2016	UK	Freq: 2 times/ week Time: NR Length: 5weeks	18	M	EG:18.1 ± 0.5 CG:18.1 ± 0.5	academy level>1 yrs	Rugby	CG: Bilateral Plyometric Training EG: Unilateral Plyometric Training	NO	10m↑

NR, Not Reported; F, Female; M, Male; EG, Experimental Group; CG, Control Group; BG, Bilateral Group; UG, Unilateral Group; B + UG, Combined Bilateral and Unilateral Group; CMJ, Countermovement Jump; SLJ, Standing Long Jump; RSI, Reactive Strength Index; 10M; 10-Meter Sprint; CMJ-R, Countermovement Jump with Right Leg; CMJ-L, Countermovement Jump with Left Leg, ↑, Significant improvement; →, No significant change; ↓, Significant decrease.

Among the included studies, one focused on badminton athletes (Wiriawan et al., 2024), one on volleyball athletes (Deng et al., 2025), one on mixed team-sport athletes (Núñez et al., 2018), five on soccer players (Stern et al., 2020; Ramírez-Campillo et al., 2015; Bettariga et al., 2023a; Bettariga et al., 2023b; Ramirez-Campillo et al., 2018), six on basketball players (Zhao et al., 2024; Cao et al., 2024; Belegišanin et al., 2025; Zhang et al., 2024; Gonzalo-Skok et al., 2019, 2017), one on judo athletes (Shi and Wu, 2019), and two on track-and-field athletes (Fisher and Wallin, 2014; Speirs et al., 2016).

A total of 491 athletes participated across all studies, including 459 males (93.4%) and 32 females (6.5%). Participant ages ranged from 10 to 27 years. With respect to competitive level, athletes were primarily classified as collegiate athletes (six studies), sub-elite athletes (four studies), national-level athletes (three studies), and local or regional team athletes (four studies). Training experience ranged from 2 to 8 years, with the majority of participants having at least three years of training experience.

In terms of intervention characteristics, the experimental groups implemented various unilateral training (PT) protocols. The intervention designs primarily included direct comparisons between unilateral and bilateral training (n=10), multi–experimental-group designs (n=3), and comparisons between unilateral training and traditional training (n=3). Regarding intervention frequency and duration, most studies implemented training twice per week (n=12), with an intervention duration of six weeks being most common (n=9). Four studies explicitly reported session duration, which ranged from 30 to 90 minutes. Overall, there was substantial consistency in intervention scheduling, with training frequencies predominantly set at two to three sessions per week and intervention durations ranging from six to eight weeks.

### Meta-analysis results

**Figure 3:**
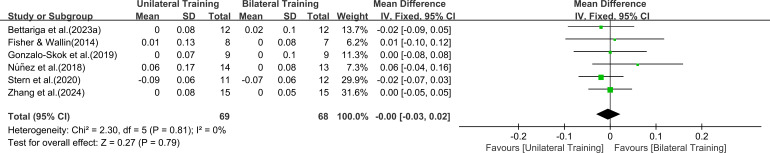
Forest plot comparing changes in CMJ between the unilateral training group and the bilateral training group in athletes.

A total of 16 studies assessing athletes’ explosive performance–related outcomes were included in this meta-analysis. The primary assessment indicators reported across the included studies consisted of CMJ, SLJ, and short-distance sprint performance. The data used for the meta-analysis are presented in **Table 2**. Among these, 10 studies (Belegišanin et al., 2025; Deng et al., 2025; Gonzalo-Skok et al., 2017, 2019; Núñez et al., 2018; Ramírez-Campillo et al., 2015; Ramirez-Campillo et al., 2018; Shi and Wu, 2019; Stern et al., 2020; Zhang et al., 2024) reported data related to vertical jump performance, involving a total of 220 athletes. The meta-analysis based on a random-effects model indicated that there was no significant difference between the unilateral training group and the bilateral training group in the improvement of CMJ performance (MD=−0.41 cm, 95% CI [−1.61,0.78], P = 0.50) (**Figure 3**).

**Figure 4:**
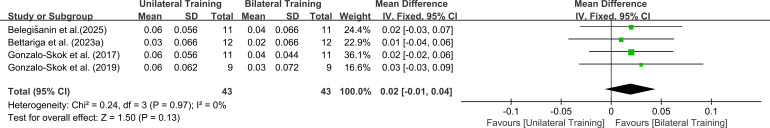
Forest plot comparing changes in 10-m sprint performance between the unilateral training group and the bilateral training group.

A total of six studies (Bettariga et al., 2023a; Fisher and Wallin, 2014; Gonzalo-Skok et al., 2019; Núñez et al., 2018; Stern et al., 2020; Zhang et al., 2024) reported data on the 10-m sprint performance, involving 139 athletes. The results of the random-effects meta-analysis indicated that there was no significant difference between the unilateral training group and the bilateral training group in the improvement of 10-m sprint performance (MD=−0.00s, 95%, CI[−0.03,0.02], p=0.79) (**Figure 4**).

**Figure 5:**
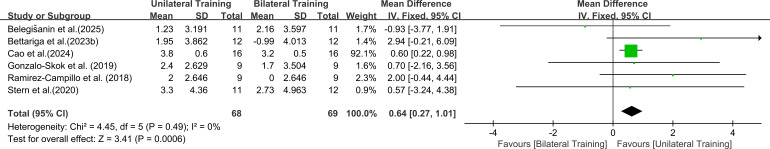
Forest plot comparing changes in 10-m sprint performance between the unilateral training group and the bilateral training group.

In total, four studies (Belegišanin et al., 2025; Bettariga et al., 2023a; Gonzalo-Skok et al., 2019, 2017) reported data on 5-m sprint performance, comprising a combined sample of 86 athletes. The results of the random-effects meta-analysis indicated that there was no significant difference between the unilateral training group and the bilateral training group in improvements in 5-m sprint performance (MD = 0.02s, 95%CI:−0.01 to 0.04,p=0.13; **Figure 5**).

In addition, two studies examined SLJ performance (Deng et al., 2025; Zhang et al., 2024), and one study assessed RSI (Belegišanin et al., 2025). With respect to SLJ performance, Deng et al. (2025) reported that both unilateral and bilateral training significantly improved SLJ performance; however, no significant difference was observed between the two training modalities (F(1,14)=0.597, p=0.455, ηp²=0.041). In contrast, Zhang et al. (2024) found that although both unilateral and bilateral training led to significant improvements in SLJ performance, a significant between-group difference was observed, favoring one training approach (t=−2.66,p=0.01). Regarding RSI, Belegišanin et al. (2025) demonstrated significant post-intervention improvements in both the unilateral and bilateral training groups, with identical relative increases of 19.6%. The corresponding effect sizes were large for both groups (d=0.95 and d=0.77, respectively).

### Additional analysis

**Figure 6:**
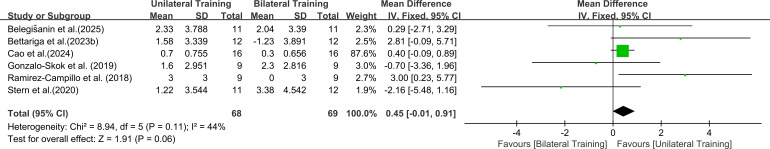
Forest plot comparing changes in CMJ-L performance between the unilateral training group and the bilateral training group.

Among the 16 studies included in this meta-analysis, six specifically investigated explosive performance in athletes (Belegišanin et al., 2025; Bettariga et al., 2023a; Cao et al., 2024; Gonzalo-Skok et al., 2019; Ramirez-Campillo et al., 2018; Stern et al., 2020), with outcome measures including CMJ-L and CMJ-R. The meta-analytic data are detailed in **Table 1**. These six CMJ studies involved 137 athletes. For CMJ-L, the random-effects model meta-analysis revealed a significant difference in improvement favoring the unilateral training group over the bilateral training group: MD = 0.64 cm, 95% CI [0.27, 1.01], P = 0.0006 (**Figure 6**).

**Figure 7:**
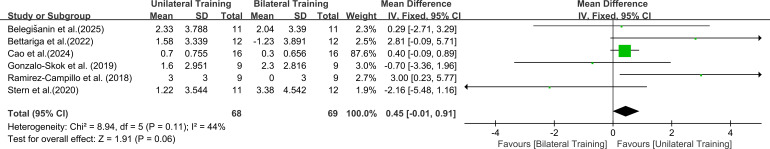
Forest plot comparing changes in CMJ-R performance between the unilateral training group and the bilateral training group.

Regarding CMJ-R performance, the results of the random-effects meta-analysis indicated no significant difference between the unilateral training group and the bilateral training group in CMJ-R improvement (MD=−0.45 cm, 95% CI: −0.01 to 0.91, p=0.06; **Figure 7**).

It should be noted that, in the studies included in this review, unilateral CMJ performance was classified according to the left leg and right leg. The participants were not categorized based on dominant and non-dominant limbs. Moreover, most of the included studies did not clearly report information regarding the participants’ limb dominance. Therefore, the present analysis was conducted based solely on anatomical laterality rather than functional dominance. Consequently, comparisons between CMJ-L and CMJ-R should not be interpreted as reflecting functional dominance effects, and the findings should be interpreted with caution.

### Adverse effects

Across the included studies, no pain, injuries, or other training-related adverse health events associated with unilateral training were reported during or after the intervention period.

## Discussion

### Effects of unilateral training on CMJ performance in athletes

The CMJ is a key indicator for assessing lower-limb explosive performance and stretch–shortening cycle efficiency, playing a central role in various sports (Haugen et al., 2020). Among the 16 studies included in this review, 10 examined the effects of unilateral training on athletes’ CMJ performance across different sports, including basketball (Belegišanin et al., 2025; Gonzalo-Skok et al., 2017, 2019; Zhang et al., 2024), football (Ramírez-Campillo et al., 2015, 2018; Stern et al., 2020), volleyball (Deng et al., 2025), badminton (Wiriawan et al., 2024), judo (Shi and Wu, 2019), and other team sports (Núñez et al., 2018). Meta-analysis results indicated no statistically significant difference between unilateral and bilateral training in improving overall CMJ performance (I²=6%,P=0.39), suggesting that the two training modalities may produce similar effects on general CMJ outcomes. Mechanistically, CMJ performance is influenced by maximal lower-limb strength, explosive force output, and stretch–shortening cycle utilization, with improvements dependent on the neuromuscular stimulus characteristics and load prescription during training (Van Hooren and Zolotarjova, 2017). Although previous studies suggest that unilateral training may offer advantages in addressing limb strength asymmetry and unilateral stability (Oliveira et al., 2013), CMJ is a bilateral coordinated movement; thus, under similar training load, intensity, and frequency, such unilateral advantages may not fully manifest in overall CMJ performance.

Among seven studies specifically assessing single-leg CMJ (involving football players: Bettariga et al., 2023a; Ramirez-Campillo et al., 2018; Stern et al., 2020; basketball players: Belegišanin et al., 2025; Cao et al., 2024; Gonzalo-Skok et al., 2019), meta-analysis showed a statistically significant effect of unilateral training on CMJ-R performance (I²=0%,P=0.006), whereas CMJ-L showed no significant change (I²=44%,P=0.06). These findings suggest that the effects of unilateral training may be more readily observed in single-leg performance. However, variations in study design and participant characteristics may influence outcomes, warranting cautious interpretation. Additionally, some studies indicate that unilateral training may enhance athletes’ change-of-direction ability and asymmetrical performance in sport-specific tasks (Tao et al., 2025).

From a training adaptation perspective, these results may be influenced by intervention duration. Improvements in CMJ ability rely on increases in strength and explosive performance and require longer-term training to optimize neuromuscular drive and joint coordination mechanisms (Thomas et al., 2017). Shorter intervention periods may be insufficient for unilateral training to confer a clear advantage in CMJ, resulting in comparable effects between unilateral and bilateral training.

Overall, current evidence suggests that unilateral and bilateral training may produce similar effects on CMJ performance in athletic populations. In practice, coaches can select training modalities based on sport-specific demands, technical requirements, and individual athlete characteristics while considering the limitations of the existing evidence. Future research should further investigate the moderating effects of training duration, load prescription, sport type, and athlete sex on different unilateral training programs to provide more robust guidance for practice.

### Effects of unilateral training on 10-m sprint performance in athletes

Among the 16 included studies, six reported data related to the 10-m sprint performance, involving basketball players (Gonzalo-Skok et al., 2019; Zhang et al., 2024), football players (Bettariga et al., 2023a; Stern et al., 2020), rugby players (Fisher and Wallin, 2014), and other team sport athletes (Núñez et al., 2018), all of whom were male. Meta-analysis results indicated no statistically significant difference between unilateral and bilateral training in improving 10-m sprint performance (I²=0%, P = 0.79), consistent with the findings of Michailidis et al. (2019) regarding the effects of unilateral versus bilateral training on 10-m sprint performance.

From a mechanistic perspective, the 10-m sprint performance primarily reflects an athlete’s acceleration capability during the initial phase, relying on maximal lower-limb strength, rapid force production, and the neuromuscular system’s ability to generate and transmit force over a short period (Marques and Izquierdo, 2014). Existing evidence indicates that both unilateral and bilateral training, when applied with appropriate load and intensity, can enhance lower-limb neuromuscular drive, thereby positively influencing short-distance acceleration performance (Speirs et al., 2016). Consequently, the two training modalities may elicit neuromuscular adaptations through different pathways, resulting in no significant differences in 10-m sprint performance.

Furthermore, the 10-m sprint performance is a technically simple task with a relatively fixed movement pattern (Brechue, 2011). Its performance primarily depends on overall lower-limb strength and instantaneous force output, with minimal demand for unilateral stability or force symmetry. In this context, bilateral training can improve limb strength and coordination, potentially producing effects comparable to unilateral training.

It is important to note that the studies included in this analysis involved only male athletes and were limited to basketball, football, rugby, and selected team sports. Although the frequency of 10-m sprint performance use and tactical demands may vary across sports, meta-analysis indicated low heterogeneity (I²=0%), suggesting a limited impact of sport type on the results and, to some extent, enhancing the robustness of the conclusions.

Current evidence suggests that unilateral and bilateral training may produce similar improvements in 10-m sprint performance among male athletes. In practice, training load and neuromuscular adaptation may be key factors influencing short-distance acceleration ability. Future research should consider including athletes from a broader range of sports and both sexes to further explore the mechanisms of unilateral training in short-distance sprinting and to provide more robust guidance for applied training.

### Effects of unilateral training on 5-m sprint performance in athletes

Among the 16 studies included in this review, four reported data on 5-m sprint performance, involving basketball players (Gonzalo-Skok et al., 2019; Belegišanin et al., 2025; Gonzalo-Skok et al., 2017) and football players (Bettariga et al., 2023a), all of whom were male. Meta-analysis results indicated no statistically significant difference between unilateral and bilateral training in improving 5-m sprint performance (I²=0%,P=0.13), consistent with previous findings on the effects of unilateral and bilateral combined training on short-distance sprint speed (Zhang et al., 2025).

From a mechanistic perspective, the 5-m sprint performance primarily reflects an athlete’s acceleration ability during the initial phase, relying on maximal instantaneous lower-limb force and rapid neuromuscular drive (Mero et al., 1992; Morin et al., 2011). Although unilateral training theoretically emphasizes single-limb strength and postural control, in force-dominant 5-m sprint performance, both unilateral and bilateral training can similarly enhance lower-limb strength and rapid neuromuscular output, which may partly explain the lack of significant differences observed in the meta-analysis. Furthermore, performance in the 5-m sprint performance is primarily determined by instantaneous horizontal propulsion and initial acceleration, with minimal reliance on single-limb strength, stability, or inter-limb coordination; thus, bilateral training may also effectively improve power output and coordinated control.

It should be noted that the included studies involved only male athletes and were limited to basketball and football players, resulting in a narrow sample representation. Nevertheless, the meta-analysis showed low heterogeneity (I²=0%), suggesting that sport type had a limited influence on the results, which enhances the reliability of the conclusions to some extent.

In summary, current evidence suggests that unilateral and bilateral training may produce similar improvements in 5-m sprint performance among male athletes. Improvements in short-distance sprint ability are likely more dependent on training intensity and lower-limb neuromuscular adaptations rather than the unilateral characteristics of the training modality. Future research should consider including athletes of different sexes, ages, and sports, with larger sample sizes, to further explore the potential mechanisms of unilateral training in short-distance sprinting and provide more robust guidance for applied training.

### Practical application

The findings of this review can provide practical guidance for coaches and athletes in training practice. Current evidence suggests that unilateral training may, to some extent, enhance athletes’ single-limb explosive performance, with potentially more pronounced effects on vertical jump ability (e.g., CMJ-R). Therefore, when the training goal is to correct lower-limb strength asymmetry, strengthen unilateral power, or meet sport-specific demands for single-limb strength, unilateral training can be considered as an optional strategy and may be combined with conventional strength or power training. In practical application, the scheduling and content of unilateral training should be tailored according to the characteristics of the sport and individual athlete differences. It should be noted that, in the studies included in this review, CMJ was categorized based on the anatomically reported left and right legs in the original publications, without explicit distinction between dominant and non-dominant limbs. Thus, caution is required when interpreting functional training effects. Future research should conduct high-quality trials to systematically assess the potential impact of unilateral training on athletes’ explosive performance under varying sports, training durations, and intensities, in order to identify its optimal application strategy.

### Limitations

This systematic review has several limitations. First, although the literature search was conducted across four databases, the coverage remains limited and may not have captured all relevant studies. Second, while the included studies involved athletes from basketball, football, volleyball, rugby, badminton, judo, and other team sports, sports primarily reliant on explosive performance, such as table tennis, golf, and boxing, were not represented. Third, due to the limited number of included studies, only selected explosive performance indicators, CMJ, CMJ-L, CMJ-R, and 10M sprint, were included in the meta-analysis, whereas other relevant indicators such as RIS and SLJ could not be analyzed quantitatively. Fourth, the majority of participants in the included studies were male athletes, with female athletes underrepresented, which limits the generalizability of the findings regarding unilateral training effects on explosive performance. Fifth, the classification of unilateral CMJ in the included studies was based solely on anatomically reported left and right legs, without distinguishing between dominant and non-dominant limbs, leaving potential effects of limb dominance on effect size unclear. Sixth, some studies provided insufficient reporting on methodological aspects such as randomization procedures, allocation concealment, and outcome assessor blinding, which may increase potential bias and affect the stability of the estimated results. Finally, according to the GRADE evidence quality assessment, most included studies were rated at moderate or lower levels of certainty, thereby reducing confidence in the reported effect estimates.

## Conclusions

The results of this study indicate that unilateral training may partially improve athletes’ CMJ-R performance, whereas no significant effects were observed for CMJ-L, overall CMJ, or 10 m sprint performance. Regarding the effects of unilateral training on athletes’ RIS and SLJ performance, existing studies have reported inconsistent findings, suggesting that current evidence is insufficient to draw definitive conclusions across all explosive performance indicators. Therefore, to more comprehensively evaluate the impact of unilateral training on athletes’ explosive capabilities, future research should consider including a broader range of relevant explosive performance indicators (e.g., RIS, SLJ) and further expand on sample characteristics (e.g., dominant vs. non-dominant limbs, sex, age) as well as study design features (e.g., methodological rigor), in order to provide more robust evidence to inform training practice.

## Data Availability

The original contributions presented in the study are included in the article/**Supplementary Material**. Further inquiries can be directed to the corresponding author.
