# Supplementary material for: Effects of unilateral training on rapid force production in athletes: a systematic review and meta-analysis
Source: Front Physiol. 2026 Apr 21;17:1805250. doi: 10.3389/fphys.2026.1805250 (PMC13139017; doi:10.3389/fphys.2026.1805250)
Supplement: Supplementary file 1 [file DataSheet1.pdf]

Year and Author	Unilateral Training	
	mean	sd
Belegišanin et al.(2025)	4.87	3.46
Deng et al.(2025)	2.83	5.47
Gonzalo-Skok et al.(2017)	2.4	4.71
Gonzalo-Skok et al. (2019)	1.4	3.7
Núñez et al.(2018)	1.56	4.9
Ramírez-Campillo et al.(2015)	2.3	4.3
Ramirez-Campillo et al. (2018)	2	4.58
Shi & Wu（2019）	3.8	4.71
Stern et al.(2020)	1.76	4.85
Zhang et al.(2024)	1.43	4.86

Year and Author		Unilateral
	mean	sd
Bettariga et al.(2023)	−0.05	0.08
Fisher & Wallin(2014)	0.01	0.13
Gonzalo-Skok et al.(2019)	−0.06	0.07
Núñez et al.(2018)	0.06	0.17
Stern et al.(2020)	-0.09	0.06
Zhang et al.(2024)	−0.11	0.08

Year and Author	mean	Unilateral sd
Belegišanin et al.(2025)	1.23	3.191
Bettariga et al.(2022)	1.95	3.862
Cao et al.(2024)	3.8	0.6
Gonzalo-Skok et al. (2019)	2.4	2.629
Ramirez-Campillo et al. (2018)	2	2.646
Stern et al.(2020)	3.3	4.36

Year and Author	mean	Unilateral sd
Belegišanin et al.(2025)	0.06	0.056
Bettariga et al. (2023)	0.03	0.066
Gonzalo-Skok et al. (2017)	0.06	0.056
Gonzalo-Skok et al. (2019)	0.06	0.062

Year and Author	mean	Unilateral sd
Belegišanin et al.(2025)	2.33	3.788
Bettariga et al.(2022)	1.58	3.339
Cao et al.(2024)	0.7	0.755
Gonzalo-Skok et al. (2019)	1.6	2.951

Ramirez-Campillo et al. (2018)	3	3
Stern et al.(2020)	1.22	3.544

	Bilateral Training		
Sample size	mean	sd	Sample size
11	3.81	5.52	11
8	0.33	4.11	8
11	1.7	5.4	11
9	0.9	4.75	9
14	1.87	4.44	13
16	5.8	2.29	12
8	3	5	8
8	2	5.39	8
11	0.99	8.07	12
15	1.47	4.28	15

l Training	Bilateral Training		
Sample size	mean	sd	Sample size
12	0.02	0.1	12
8	−0.07	0.08	7
9	−0.01	0.1	9
14	0	0.08	13
11	-0.07	0.06	12
15	−0.07	0.05	15

l Training	Bilateral Training		
Sample size	mean	sd	Sample size
11	2.16	3.597	11
12	-0.99	4.013	12
16	3.2	0.5	16
9	1.7	3.504	9
9	0	2.646	9
11	2.73	4.963	12

l Training	Bilateral Training		
Sample size	mean	sd	Sample size
11	0.04	0.066	11
12	0.02	0.066	12
11	0.04	0.044	11
9	0.03	0.072	9

l Training	Bilateral Training		
Sample size	mean	sd	Sample size
11	2.04	3.39	11
12	-1.23	3.891	12
16	0.3	0.656	16
9	2.3	2.816	9

9	0	3	9
11	3.38	4.542	12
