# Supplementary figures and images for: Effects of unilateral training on rapid force production in athletes: a systematic review and meta-analysis
Source: Front Physiol. 2026 Apr 21;17:1805250. doi: 10.3389/fphys.2026.1805250 (PMC13139017; doi:10.3389/fphys.2026.1805250)

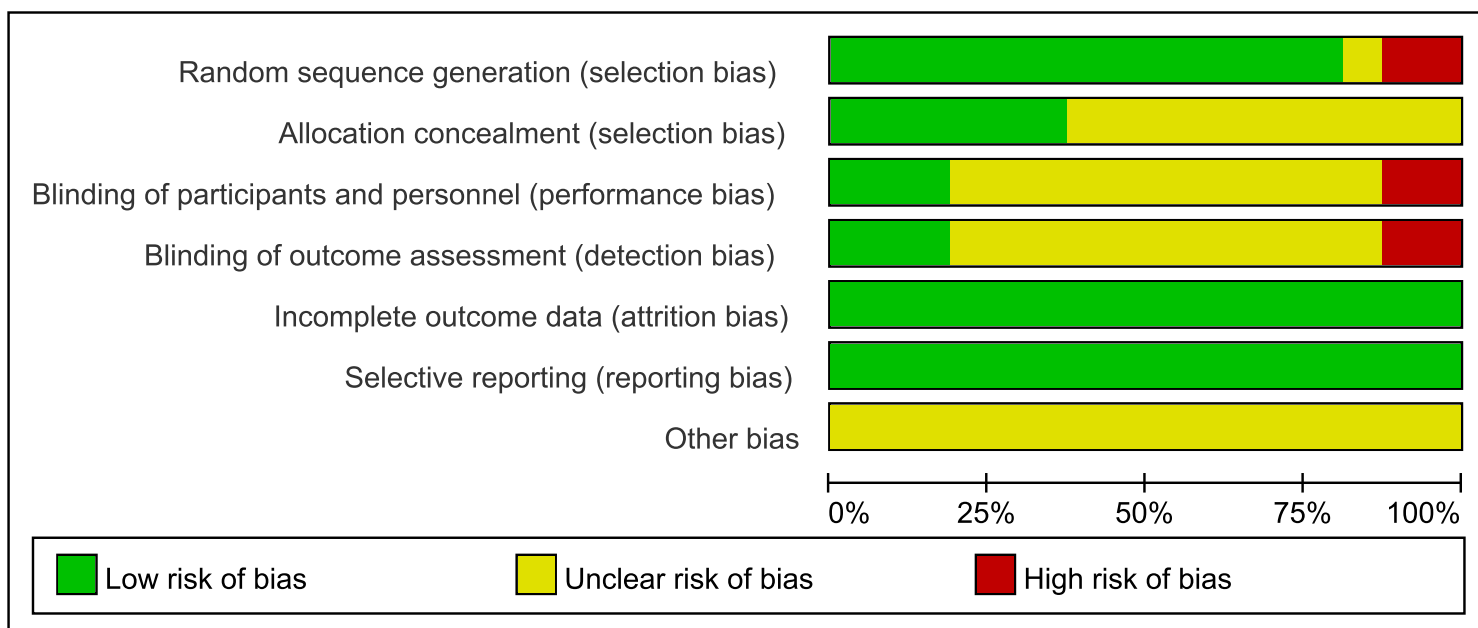

Supplement: Supplementary file 2 [file Image1.pdf]

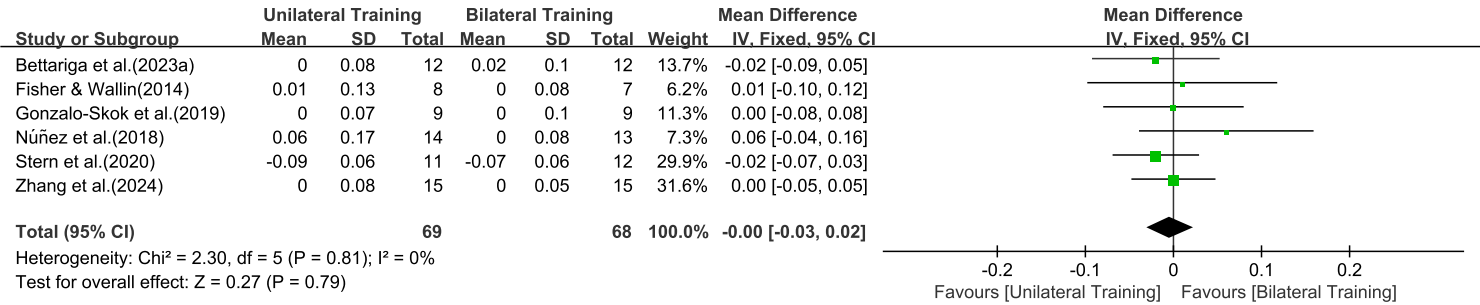

Supplement: Supplementary file 4 [file Image3.pdf]

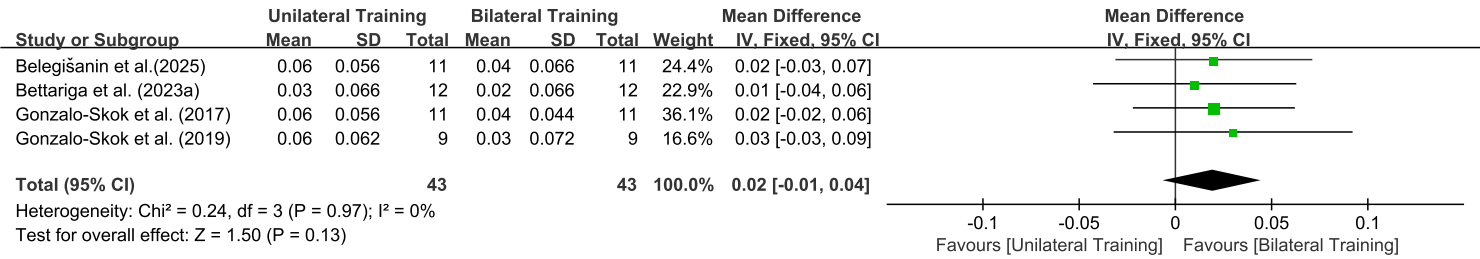

Supplement: Supplementary file 5 [file Image4.pdf]

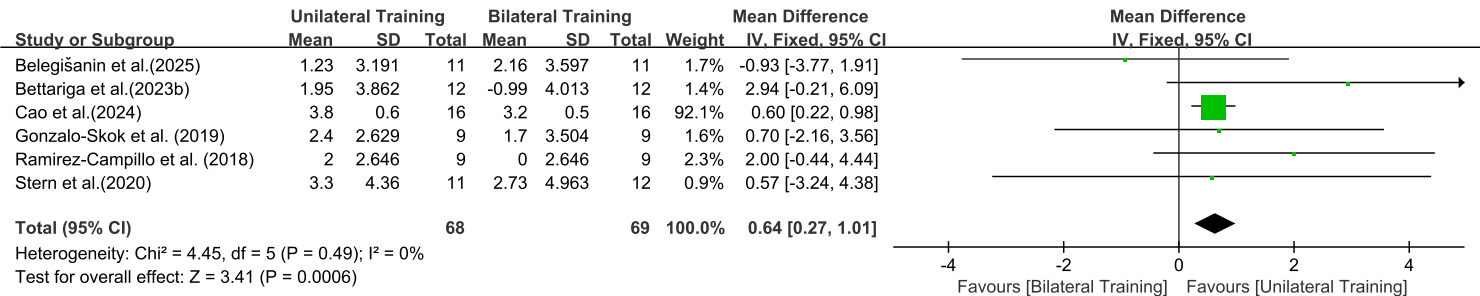

Supplement: Supplementary file 6 [file Image5.pdf]

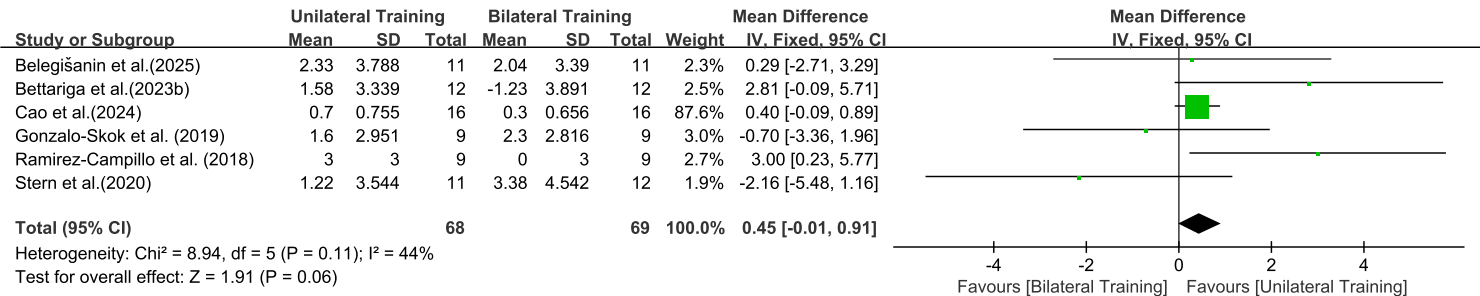

Supplement: Supplementary file 7 [file Image6.pdf]

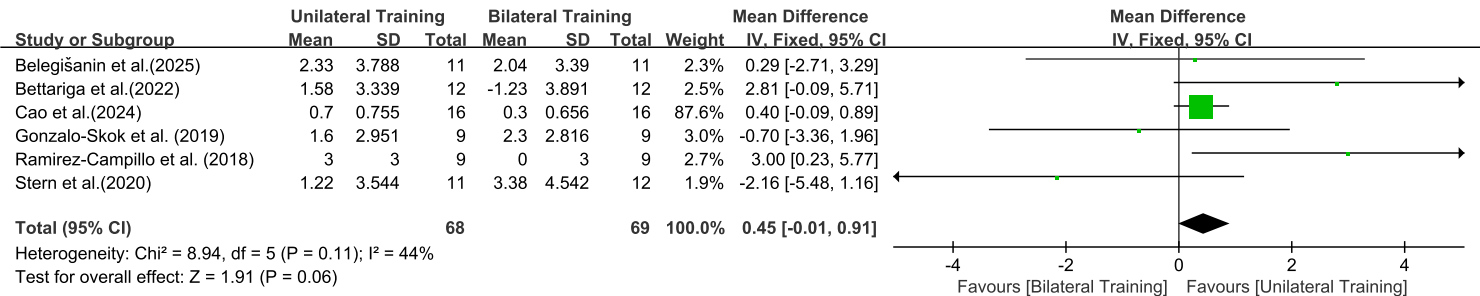

Supplement: Supplementary file 8 [file Image7.pdf]
