# Supplementary material for: Effects of unilateral training on rapid force production in athletes: a systematic review and meta-analysis
Source: Front Physiol. 2026 Apr 21;17:1805250. doi: 10.3389/fphys.2026.1805250 (PMC13139017; doi:10.3389/fphys.2026.1805250)
Supplement: Supplementary file 3 [file Image2.pdf]

	Random sequence generation (selection bias)	Allocation concealment (selection bias)	Blinding of participants and personnel (performance bias)	Blinding of outcome assessment (detection bias)	Incomplete outcome data (attrition bias)	Selective reporting (reporting bias)	Other bias
Belegišanin et al.(2025)	+	?	−	−	+	+	?
Bettariga et al.(2022b)	+	+	?	?	+	+	?
Bettariga et al.(2023a)	+	+	?	?	+	+	?
Cao et al.(2024)	+	+	+	+	+	+	?
Deng et al.(2025)	+	?	?	?	+	+	?
Fisher & Wallin(2014)	+	+	?	?	+	+	?
Gonzalo-Skok et al.(2017)	?	?	?	?	+	+	?
Gonzalo-Skok et al. (2019)	+	?	?	?	+	+	?
Núñez et al.(2018)	−	?	?	?	+	+	?
Ramírez-Campillo et al.(2015)	+	?	?	?	+	+	?
Ramirez-Campillo et al. (2018)	+	?	+	+	+	+	?
Shi & Wu（2019）	−	?	?	?	+	+	?
Speirs et al.(2016)	+	+	?	?	+	+	?
Stern et al.(2020)	+	?	?	?	+	+	?
Zhang et al.(2024)	+	?	−	−	+	+	?
Zhao et al.(2024)	+	+	+	+	+	+	?
