# Supplementary material for: Effects of unilateral training on rapid force production in athletes: a systematic review and meta-analysis
Source: Front Physiol. 2026 Apr 21;17:1805250. doi: 10.3389/fphys.2026.1805250 (PMC13139017; doi:10.3389/fphys.2026.1805250)
Supplement: Supplementary file 9 [file Table1.pdf]

TABLE 1 PEDro scale scores of the included studies.

Study name	N°1	N°2	N°3	N°4	N°5	N°6	N°7	N°8	N°10	N°11	Total*	Study quality
Deng et al.(2025)	1	1	0	1	0	0	0	0	1	1	5	Moderate
Núñez et al.(2018)	1	1	0	1	0	0	0	1	1	1	6	High
Stern et al.(2020)	1	1	0	1	0	0	0	1	1	1	6	High
Ramírez-Campillo et al.(2015)	1	1	0	1	0	0	0	1	1	1	6	High
Bettariga et al.(2023)	1	1	1	1	0	0	0	1	1	1	6	High
Zhao et al.(2024)	1	1	1	1	0	0	1	1	1	1	7	High
Cao et al.(2024)	1	1	1	1	0	0	1	1	1	1	7	High
Bettariga et al.(2023)	1	1	1	1	0	0	0	0	1	1	5	Moderate
Drouzas et al.2020）	1	1	0	0	0	0	0	0	1	1	3	Low
Belegišanin et al.(2025)	1	1	0	0	0	0	0	1	1	1	4	Moderate
Gonzalo-Skok et al.(2022)	1	1	0	0	0	0	0	1	1	1	3	Low
Zhang et al.(2024)	1	1	0	0	0	0	0	1	1	1	4	Moderate
Ramirez-Campillo et al.(2018）	1	1	0	0	0	0	1	1	1	1	5	Moderate
Gonzalo-Skok et al.(2019)	1	1	0	1	0	0	1	0	1	1	5	Moderate
Shi & Wu(2019）	1	1	0	1	0	0	0	1	1	1	5	Moderate
Fisher & Wallin(2014)	1	1	1	1	0	0	0	0	1	1	5	Moderate
Gonzalo-Skok et al.(2017)	1	1	0	1	0	0	0	0	1	1	4	Moderate
Speirs et al.(2016)	1	1	0	1	0	1	1	0	1	1	7	High
