# Supplementary material for: Effects of unilateral training on rapid force production in athletes: a systematic review and meta-analysis
Source: Front Physiol. 2026 Apr 21;17:1805250. doi: 10.3389/fphys.2026.1805250 (PMC13139017; doi:10.3389/fphys.2026.1805250)
Supplement: Supplementary file 10 [file Table2.pdf]

Table 2. Characteristics of participants and PT interventions in the included studies.										
**Study**	**Country**	**Intervention**	**N**	**Sex**	**Age**	**Level/ Experience**	**Sport**	**Comparison**	**Replace**	**Outcome (s)**
Deng et al.(2025)	China	Freq: 2 times/ week Time: NR Length: 8 weeks	16	F	EG1:18.75±0.707<br>EG2:19.25±0.707	Collegiate<br>> 5-yrs	Volleyball	EG1:Unilateral Training Group<br>EG2:Bilateral Training Group	NO	CMJ↑<br>SLJ↑
Núñez et al.(2018)	Spain	Freq: 2 times/ week Time: NR Length: 6 weeks	27	M	EG1:22.8 ± 2.9<br>EG2:22.6 ± 2.7	Collegiate<br>NR	Team sports	EG1:Unilateral Lunge Squat Group<br>EG2:Bilateral Lunge Squat Group	NO	CMJ→
Stern et al.(2020)	UK	Freq: 2 times/ week Time: NR Length: 6 weeks	23	M	17.6±1.2	Local soccer Team<br>> 2-yrs	Soccer	EG1:Bulgarian split squat, single-leg vertical jump, single-leg drop jump, single-leg standing long jump.<br>EG2:Back squat, bilateral vertical jump, bilateral drop jump, bilateral standing long jump.	NO	CMJ↓<br>RSI→<br>10M↑
Ramírez-Campillo et al.(2015)	Chile	Freq: 2 times/ week Time: NR Length: 6 weeks	54	M	CG：1.2± 2.4<br>BG：11.0 ± 2.0<br>UG：11.6 ± 1.7<br>B + UG：11.6 ± 2.7	Sub-elite 3–4 yrs	Soccer	CG:Conventional Soccer Training<br>EG1:Unilateral Concentric-Concentric Jump Training Group<br>EG2:Bilateral Concentric-Concentric Jump Training Group<br>EG3:Combined Bilateral+Unilateral Jump Training Group	YES	CMJA ↑<br>CMJ-R ↑<br>CMJ-L ↑

Bettariga et al.(2023a)	UK	Freq: 2 times/ week Time: 60min Length: 6 weeks	24	M	25.4±4.9	Sub-elite >3 yrs	Soccer	CG:Conventional Training<br>EG:Unilateral Strength+Plyometric Jump Training	NO	5 m→<br>10 m↑
Zhao et al.(2024)	China	Freq: 3-4 times/ week Time: NR Length: 8 weeks	75	M	**Upjt：EG1：**16.2 ± 0.6<br>**bPJT：**EG2:16.4 ± 0.6<br>**Control：**16.4 ± 0.5	Local Basketball Players Team >3 yrs	Basketball	CG:Conventional Training<br>EG1:Unilateral Plyometric Jump Training Group<br>EG2:Bilateral Plyometric Jump Training Group	NO	CMJ↑
Cao et al.(2024)	China	Freq: 2 times/ week Time: NR Length: 8weeks	66	M	UT:EG1 ∶15.9 ± 0.9<br>BT:EG2 ∶ 6.3 ± 0.8<br>UBT:EG3: ∶ 16.2 ± 0.7<br>Control ∶CG:16.1 ± 0.8	Local Basketball Players Team >2 yrs	Basketball	CG:Conventional Training<br>EG1:Unilateral Plyometric Training Group<br>EG2:Bilateral Plyometric Training Group<br>EG3:Combined Unilateral+Bilateral Plyometric Training Group	NO	CMJ-L↑<br>CMJ-R↑
Bettariga et al.(2022b)	Italy	Freq: 2 times/ week Time: NR Length: 6weeks	24	M	EG:27.0±4.8<br>CG:23.8±4.8	Local soccer Team >3 yrs	Soccer	CG:Conventional Training<br>EG:Unilateral Strength+Power Training	NO	CMJ-R ↑<br>CMJ-L↑<br>SLBJ-R ↑<br>SLBJ-L↑
Belegišanin et al.(2025)	Serbia	Freq: 4 times/ week Time: 90min	22	M	EG(UPT):15.5 ± 0.5<br>EG2(BPT):15.2 ±	Local soccer Team >4 yrs	Basketball	EG1:Unilateral Flywheel Strength Training Group<br>EG2:Bilateral Flywheel Strength Training	NO	RSI↑<br>CMJ↑<br>5m↑

		Length: 6weeks			0.4			Group		
Zhang et al.(2024)	China	Freq: 3 times/ week Time: 30min Length: 10weeks	30	M	EG：20.9 ± 1.1<br>CG：20.9 ± 0.9	Collegiate >4 yrs 4.1 ± 0.8 yrs	Basketball	CG:Conventional Training<br>EG:Unilateral Combined Training	NO	CMJ ↑<br>SLJ↑<br>10m→
Ramirez-Campillo et al. (2018)	Spain	Freq: 2 times/ week Time: NR Length: 8weeks	18	M	EG（UG）：17.3 ± 1.1<br>CG（TG）：17.6 ± 0.5	Collegiate >8 yrs	Soccer	CG:Conventional Training<br>EG:Unilateral Plyometric Training Group	NO	CMJ↓
Gonzalo-Skok et al. (2019)	Spain	Freq: 2 times/ week Time: 35-40min Length: 6weeks	18	M	13.2 ± 0.7	National-level Athlete	Basketball	CG:Bilateral Vertical Power Training<br>EG:Unilateral Horizontal Power Training	NO	CMJ↑<br>5m↑<br>10m↑
Shi & Wu（2019）	China	Freq: 3 times/ week Time: NR Length: 10weeks	16	F	20. 5	National-level Athlete >5yrs	Judo	CG:Bilateral Lower-Limb Resistance Training<br>EG:Unilateral Limb Resistance Training	NO	CMJ ↑
Fisher & Wallin(2014)	UK	Freq: 2 times/ week Time: NR Length:	15	M	CG（TG）：20.14 ±1.77<br>EG（UG）：19.8 ±1.49	collegiate level	Rugby	CG:Bilateral Lower-Limb Resistance Training<br>EG:Unilateral Limb Resistance Training	NO	10m↓

		6weeks								
Gonzalo-Skok et al.(2017)	Spain	Freq: 2 times/ week Time: NR Length: 6weeks	22	M	EG（UG）：：16.8±1.7<br>CG（TG）：16.7±1.7	National-level Athlete >2 yrs	Basketball	CG:Bilateral Lower-Limb Resistance Training Group<br>EG:Unilateral Limb Resistance Training Group	NO	CMJ→<br>5 m→
Speirs et al.(2016)	UK	Freq: 2 times/ week Time: NR Length: 5weeks	18	M	EG：18.1 ±0.5<br>CG：18.1±0.5	academy level>1 yrs	Rugby	CG:Bilateral Plyometric Training<br>EG:Unilateral Plyometric Training	NO	10m↑
